# Dissecting the phenomenon of suicidality in patients with obsessive-compulsive disorder: A descriptive psychopathological study

**DOI:** 10.1192/j.eurpsy.2025.270

**Published:** 2025-08-26

**Authors:** Y. A. Ferrao, J. V. Bueno Ferrão, J. R. da Silva, T. Yamaguchi

**Affiliations:** 1 Psychiatry, UFCSPA; 2 Psychiatry, HMIPV; 3 Medicine, PUCRS; 4 Medicine, UFCSPA, Porto Alegre, Brazil

## Abstract

**Introduction:**

There are conflicting and inconclusive results regarding suicidality in patients with obsessive-compulsive disorder (OCD).

**Objectives:**

To describe the different psychopathological aspects of suicidality (suicidal ideation, suicide attempts, completed suicide, family history, etc.) in OCD.

**Methods:**

In this case-control study we compared 114 patients with OCD and 127 controls (no OCD general hospital inpatients). The psychopathological aspects of suicidality were obtained through a 24 questions specific suicidality inventory (DETEC-S). Other instruments/data (Beck Depression and Anxiety Inventories, family history of psychiatric disorders, symptom severity scale, and psychiatric comorbidities) will be described in another study.

**Results:**

Patients with OCD reported: less will to live (p=0.005); greater will to die (p<0.001); more reasons to die (p=0.022); greater lifetime history of suicidal ideation (SI) (p<0.001); accept the idea of dying (p<0.001); admit death as an escape from problems (p<0.001); suicidal plan in the past (p=0.019); admit courage and ability to commit suicide (p=0.003); no organization for the afterlife (p=0.021); have already talked to other people about SI (p<0.001); access to a lethal method (p=0.047); higher rates of family members, close people or friends who have already attempted suicide (p=0.008). They did not differ from the controls: would save themselves in case of an accident; current intention to commit suicide; able to control suicidal desire; would not commit suicide because of family, friends or religion; absence of current suicidal plan; suicidal desire or plan in the near future; writing a suicide note; prevalence of lifetime suicide attemp (SA); when they had SA, there was no need for medical care; and suicide success rates of family members, close people or friends. There was no significant difference in the total DETECT-S score (p=0.086). There was a moderate and direct correlation of suicidality scores with: the current severity of OCD and (r=0.32; p=0.001), especially at the expense of the severity of compulsions (r=0.35; p<0.001); and with the severity of depressive symptoms (r=0.43; p<0.001); average rate of lifetime improvement of symptoms (r=0.36; p=0.007). We found a moderate and indirect correlation with total quality of life scores (r=-0.47; p<0.001), especially at the expense of aspects related to the physical (r=-0.47), psychological (-0.47) and environmental (r=-0.42) domains (all with p<0.001).

**Conclusions:**

Suicidality appears to be markedly present in patients with OCD (of the 24 psychopathological aspects of the DETECT-S questionnaire, patients had higher scores in 13 items) even when compared to patients admitted to a general hospital. A detailed approach to suicidality in patients with OCD may help in therapeutic planning and reduce patient suffering.

**Disclosure of Interest:**

None Declared

